# Optimization of Proportions of Alkali-Activated Slag–Fly Ash-Based Cemented Tailings Backfill and Its Strength Characteristics and Microstructure under Combined Action of Dry–Wet and Freeze–Thaw Cycles

**DOI:** 10.3390/ma17204945

**Published:** 2024-10-10

**Authors:** Jianlin Hu, Zhipeng Meng, Tongtong Gao, Shaohui Dong, Peng Ni, Zhilin Li, Wenlong Yang, Kai Wang

**Affiliations:** 1 Key Laboratory of Urban Security and Disaster Engineering of Ministry of Education, Beijing University of Technology, Beijing 100124, China; 2 College of Civil Engineering, Hebei University of Architecture, Zhangjiakou 075000, China; 15532395383@163.com (Z.M.); 18234813517@163.com (T.G.); 15033039814@163.com (S.D.); lizhilin001216@163.com (Z.L.); yangwenlong9658@163.com (W.Y.); 15151825930@163.com (K.W.); 3 Hebei Innovation Center of Transportation Infrastructure in Cold Region, Zhangjiakou 075000, China; 4 School of Aerospace, Mechanical and Mechatronic Engineering, The University of Sydney, Sydney, NSW 2006, Australia

**Keywords:** alkali activated, cemented tailings backfill, response surface methodology, dry–wet and freeze–thaw cycles, microstructure

## Abstract

To enhance the application of alkali-activated materials in mine filling, cemented tailings backfill was prepared using slag, fly ash, sodium silicate, and NaOH as primary constituents. The effects of the raw material type and dosage on the backfill were examined through a single-factor experiment. Additionally, response surface methodology (RSM) was utilized to optimize the mixing ratios of the backfill, with a focus on fluidity and compressive strength as key objectives. The evolution of backfill quality and compressive strength under the combined effects of dry–wet and freeze–thaw (DW-FT) cycles was analyzed. The hydration products, microstructure, and pore characteristics of the specimens were analyzed using X-ray diffraction (XRD), scanning electron microscopy with energy dispersive spectroscopy (SEM-EDS), and nitrogen adsorption tests (NATs) across varying cycles. The results demonstrate that the optimal backfill composition includes 47.8% fly ash, 6.10% alkali equivalent, and a 1.44 sodium silicate modulus. The macroscopic behavior of the backfill under DW-FT coupling followed this progression: pore initiation → pore expansion → crack formation → crack propagation → structural damage. After a minor initial increase, the backfill strength steadily decreased. Microscopic analysis revealed that the decline in internal cementation products and the deterioration of pore structure were the primary causes of this strength reduction. Thus, the DW-FT coupling can cause significant erosion of the backfill. The technical solutions presented in this paper offer a reference for solid waste utilization and provide valuable insights into the durability of backfill under DW-FT coupling.

## 1. Introduction

Mineral mining has driven the rapid development of industry, but it has also inevitably resulted in numerous goafs and solid wastes [1]. Large-scale goafs can compromise mine safety and are likely to trigger geological hazards such as mudslides [2,3]. Backfilling mining is an environmentally friendly technology that utilizes solid waste to fill goafs and has gradually become one of the most widely adopted methods in modern mining [4,5]. Currently, cement is frequently utilized as a binder in backfilling mining due to its established technology [6]. However, when cement is utilized as the binding material in mine filling, its cost constitutes 60–80% of the total expenses [7]. Furthermore, the cement production process releases significant amounts of CO_2_. According to statistics, annual CO_2_ emissions from cement production in China represent 5–8% of total global carbon emissions [8,9]. Under China’s Dual Carbon target strategy, traditional high energy-consuming industries must transition to greener practices. Consequently, there is an urgent need to investigate low-carbon, cost-effective cementitious material suitable for mine filling. Alkali-activated materials (AAM) are an emerging binder primarily made from industrial solid waste, with the CO_2_ emissions during production being less than 40% of those from silicate cement. Additionally, AAM exhibit excellent mechanical properties and durability [10,11]. Meanwhile, the use of AAM can help eliminate significant amounts of solid waste generated by the steel, electricity, and other industries, contributing to the sustainable development of the economy and society [12].

Many researchers have conducted extensive studies on the strength characteristics of alkali-activated materials. Wang et al. [13] developed a novel cementitious material by activating electrolytic manganese slag, slag, and red mud using NaOH, and also produced cemented tailings backfill with tailings aggregate. The study showed that when the dosage of electrolytic manganese slag was 20%, the cemented backfill achieved optimal performance, with a 28-day strength reaching 5.45 MPa. Liu et al. [14] selected slag micropowder and fly ash as aluminosilicate precursors and systematically studied the effects of one- and two-component activators, including five alkaline salts and triethanolamine, on the dynamic strength of the backfill. The results indicated that the binary activator, comprising 4% Na_2_SiO_3_ and 4% NaAlO_2_, significantly increased the 3-day and 7-day strength. Li et al. [15] used NaOH, phosphogypsum, high-magnesium nickel slag, and granulated blast furnace slag as raw materials for alkali-activated cementitious materials, with tailings as the aggregate for backfill preparation. The optimal backfill performance was achieved with a slag dosage of 30% and an alkali dosage of 6%. Sun et al. [16] prepared alkali-activated backfill using gangue and fly ash as precursors. The 28-day compressive strength reached 2.16 MPa with a mass concentration of 79.65%, a gangue content of 57.19%, and a fly ash content of 15.67%. Additionally, numerous researchers have studied the mix ratio design of backfill. Huang et al. [17] studied the effects of slurry concentration, cement content, and waste rock admixture on backfill strength, using a multi-objective function method to design the cemented neutralization slag backfill ratio. He et al. [18] improved the random forest model using the beetle search algorithm and designed the backfill proportion based on this enhanced model. The results demonstrated that the hybrid machine learning model effectively predicts compressive strength and introduces a new concept for mix ratio design.

Numerous researchers have investigated the durability of backfill, examining its microstructural characteristics through tests such as XRD and SEM. Wang et al. [19] investigated the effects of dry–wet cycles and curing ages on the mechanical properties of specimens using a series of compression, tensile, and shear tests. The SEM analysis revealed that as the number of cycles increased, the internal structure of the specimens gradually became looser, with more pronounced cracks and pores. Liu et al. [20] investigated the variation of unconfined compressive strength in backfill material subjected to dry–wet cycles and salt corrosion, establishing a life prediction model for the backfill material based on the Wiener stochastic process. The study revealed that the hydration products resulting from the Cl^−^ attack were primarily short columnar gypsum. Song et al. [21] introduced the principle of energy conservation by examining the evolution of macroscopic mechanical strength and microstructure and thoroughly investigated the mechanism of specimen destabilization influenced by dry–wet cycles. The porosity changes of the backfill were investigated using nuclear magnetic resonance (NMR) tests. Results indicated that the porosity of the specimens initially decreased and subsequently increased with the number of cycles. Ding et al. [22] analyzed the deterioration mechanism of freeze–thaw damage in backfill material using resistivity and ultrasonic testing technologies; this study provides a theoretical basis for the non-destructive and efficient measurement of backfill strength.

Numerous studies on the strength characteristics, mixing ratio design, durability, and microstructure of alkali-activated materials have significantly enhanced their application in mine filling. However, fewer studies have examined the effects of interactions between various solid wastes and alkali activators on backfill properties. Additionally, the macroscopic and fine-scale mechanisms of alkali-activated backfill materials under DW-FT cycling remain unclear. To facilitate the broader application of alkali-activated materials in mine filling, cemented tailings backfill was prepared using slag, fly ash, sodium silicate, and NaOH as primary raw materials. RSM was employed to investigate the effects of interactions among the fly ash dosage, alkali equivalent, and sodium silicate modulus on backfill properties. A response surface model was developed to evaluate the fluidity and 28-day compressive strength of the backfill. Finally, XRD, SEM-EDS, and NATs were conducted to elucidate the micro-mechanisms of the backfill under the combined action of DW-FT cycles.

## 2. Materials and Methods

### 2.1. Materials

#### 2.1.1. Solid Waste Raw Materials

The solid waste raw materials consist of slag, fly ash, and iron tailings. Slag and fly ash serve as silica-alumina feedstocks with specific volcanic ash activity, while iron tailings function as aggregates. The slag used in the tests was S95 slag powder, with a specific surface area of 477 $m^{2}$/kg. The fly ash used was Grade II, with a specific surface area of 428 $m^{2}$/kg. Both slag and fly ash were sourced from Hebei Lingshou Chuangbo Mineral Products Co., Ltd. (Shijiazhuang, China). The iron tailings were obtained from a tailings depot in northern Hebei and consisted of fine particles with black and blue coloration.

**Figure 1:**
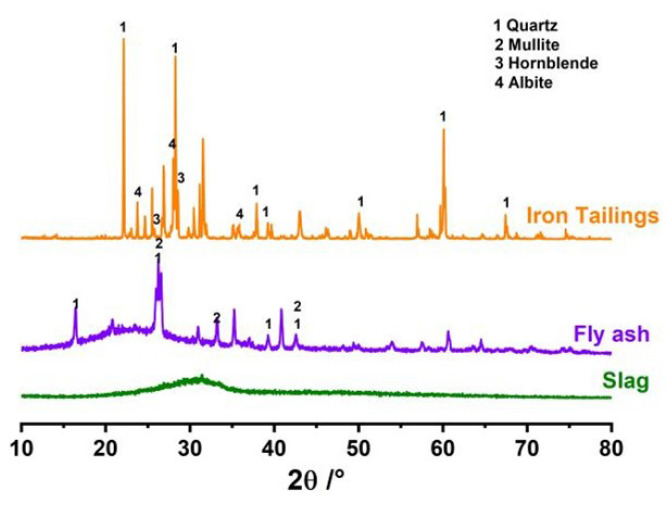
XRD pattern of raw material.

**Figure 2:**
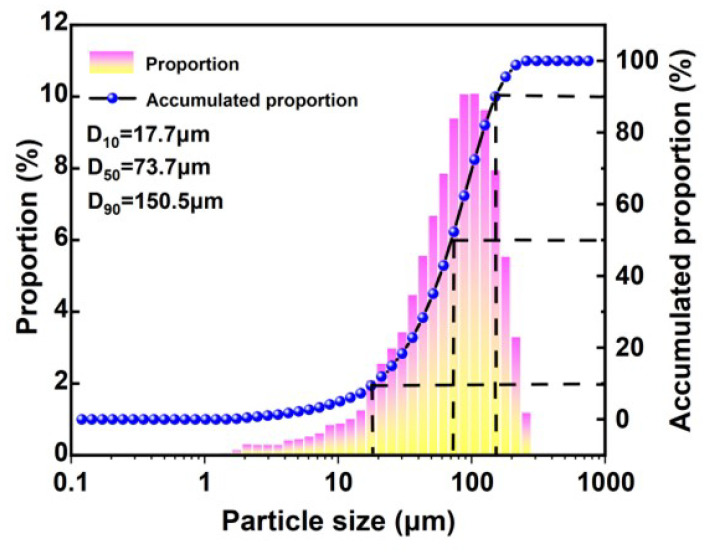
Particle size distribution curve of iron tailings.

**Figure 3:**
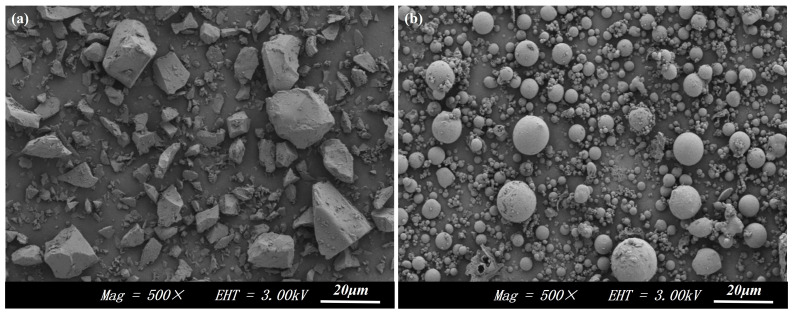
Microscopic morphology of (**a**) slag and (**b**) fly ash.

The chemical composition of the solid waste raw materials was analyzed using X-ray fluorescence (XRF), and the results are presented in Table 1. The main component of slag is Cao, its alkalinity coefficient M_0_ = 1.12 (>1), and its quality coefficient K = 2.12 (>1.8), which belongs to alkalinity slag with high potential activity [23]. Additionally, fly ash contains significant active substances with volcanic ash effects, with a total Si and Al content reaching 80%, demonstrating its potential in producing cementitious materials [24]. The mineral composition of the solid waste raw materials was analyzed using XRD, with results presented in Figure 1. Figure 1 shows that the XRD pattern of slag exhibits broad and diffuse diffraction peaks; the main mineral phases of fly ash include quartz, mullite, and amorphous glass. The particle size distribution of the iron tailings was analyzed using a laser particle size analyzer [14], with results presented in Figure 2. The $D_{10}$, $D_{50}$, and $D_{90}$ values for the iron tailings were 17.7 μm, 73.7 μm, and 150.5 μm, respectively. The microscopic morphology of slag and fly ash was examined using a scanning electron microscope, with results presented in Figure 3. Figure 3a illustrates that slag predominantly consists of irregular, lumpy particles exhibiting considerable variation in particle size. In contrast, Figure 3b shows that the majority of fly ash consists of spherical, regular particles with smooth surfaces.

#### 2.1.2. Alkali Activator

Sodium silicate and NaOH were selected as alkali activators, with the moduli being adjusted by varying the amounts of NaOH added to the sodium silicate. The sodium silicate used was produced by Zhengzhou Quancheng Industry Co., Ltd. (Zhengzhou, China), containing 26.2% SiO_2_, 8.3% Na_2_O, and 65.5% water. NaOH, which is analytically pure solid (purity ≥ 96%), was supplied by Xilong Science and Chemical Industry.

The alkali activator solution was prepared by mixing sodium silicate and NaOH with water, stirring until the NaOH completely dissolved, and allowing the mixture to stand for 12 h. Before testing, it was ensured that the solution was mixed thoroughly.

#### 2.1.3. Sulfate Activator

Desulfurization gypsum was selected as a sulfate activator. In an alkaline environment, desulfurization gypsum serves as an active sulfate activator, supplying significant amounts of ${\mathrm{SO}}_{4}^{2-}$ for the hydration reaction and promoting the formation of additional AFt.

### 2.2. Specimen Preparation

**Figure 4:**
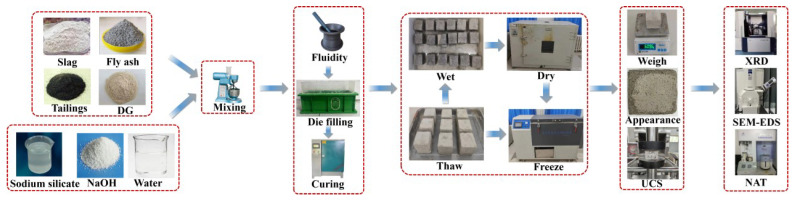
Specimen preparation and testing flow chart.

First, different ratios of solid waste raw materials and desulfurization gypsum were thoroughly mixed using a planetary mixer. After stirring for 60 s, the prepared alkali activator solution and additional water were added. The mixture was stirred again for 4 min, and then the resulting slurry was poured into truncated cone molds for the fluidity test. The fresh mud was then carefully poured into a triple mold measuring 70.7 mm × 70.7 mm × 70.7 mm. The specimen was removed from the mold after 24 h. Finally, the specimens were cured at 20 °C and 95% humidity until they reached the designated age. Specimens cured for 28 days underwent DW-FT cycle tests. After each cycle, changes in the specimen quality, appearance, and compressive strength were measured, followed by microscopic analyses including XRD, SEM-EDS, and NATs. Figure 4 shows the sample preparation and testing process.

### 2.3. Design of Experiment

#### 2.3.1. Design of Single-Factor Experiments

Fly ash dosage was designated as factor A, alkali equivalent as factor B, and sodium silicate modulus as factor C. A single-factor test was performed to examine the effects of these three factors on the working and mechanical properties of the backfill. In the test, the dosages of desulfurization gypsum (6% of the mass of slag and fly ash), mass concentration (79%), and binder–aggregate ratio (1:5) were maintained constant. The design of the single-factor experiments is illustrated in Table 2.

#### 2.3.2. Design of Experiments Using Response Surface Methodology

RSM combines experimental design with mathematical modeling for optimization [25,26]. This study utilized the Box–Behnken Design (BBD) to investigate the effects of the fly ash dosage, alkali equivalent, and sodium silicate modulus on the properties of backfill. The influencing factors and their levels are summarized in Table 3.

The BBD results were statistically analyzed using Design-Expert 13 software. The significance of each factor on the response value was evaluated using the *p*-value: *p* < 0.01 indicates a highly significant effect; 0.01 < *p* < 0.05 indicates a significant effect; and *p* > 0.05 indicates no significant effect [27].

#### 2.3.3. Design of Dry–Wet and Freeze–Thaw Cycle Experiments

The backfill specimens were prepared according to the optimal ratio, cured under standard conditions for 28 days, and then subjected to DW-FT cycle experiments. First, the specimen is placed in a 20 °C constant temperature water bath for 12 h, removed for 15 min, and then dried in a 30 °C oven for 6 h. After returning to room temperature, the specimen is frozen at −25 °C in a low-temperature chamber for 12 h and then thawed in a 25 °C ambient environment for 6 hours, completing one DW-FT cycle. After 0, 1, 3, 5, 7, 9, 11, 13, and 15 DW-FT cycles, the appearance, mass, and compressive strength of the specimens were recorded, and the microstructure of selected specimens was analyzed.

### 2.4. Test Methods

#### 2.4.1. Compressive Strength and Fluidity Tests

The uniaxial compressive strength test was conducted according to GB/T 17671-2021 [28] at a loading speed of 1 mm/min, using the average strength of three parallel specimens. The fluidity test was performed in accordance with GB/T 2419-2005 [29], using a φ 70 × 100 × 60 mm truncated cone mold to measure the slurry’s fluidity.

#### 2.4.2. Microscopic Tests

Microscopic tests were performed on specimens at different cycle numbers. The central fragments were immersed in anhydrous ethanol for 24 h to terminate hydration [30]. Fresh sections of the samples were prepared for SEM-EDS analysis, while the remaining fragments were ground to 80 μm and analyzed using XRD-EMPYREAN. Finally, a small block with a volume of approximately 1 cm^3^ and a mass of 1 to 2 g was prepared for the NAT.

The SEM test was conducted using the Sigma300 scanning electron microscope manufactured by Zeiss, while the EDS test utilized the Octance Plus X-ray spectrometer produced by EDAX, USA (Mahawa, NJ, USA). Prior to scanning, the specimen was treated with a gold spray. The XRD test was performed using the Empyrean X-ray diffractometer manufactured by the Dutch company Parnack, with a scanning range of 5° to 80° and a scanning speed of 10°/min. The NAT was performed using a 3H-2000PM1 (Beishide Instrument, Beijing, China) high-performance pore size analyzer, measuring parameters such as pore size distribution, pore volume, and pore diameter, according to the Barret–Joyner–Halenda (BJH) method [31].

## 3. Results and Discussion

### 3.1. Results of Single-Factor Experiments

#### 3.1.1. Fly Ash Dosage

**Figure 5:**
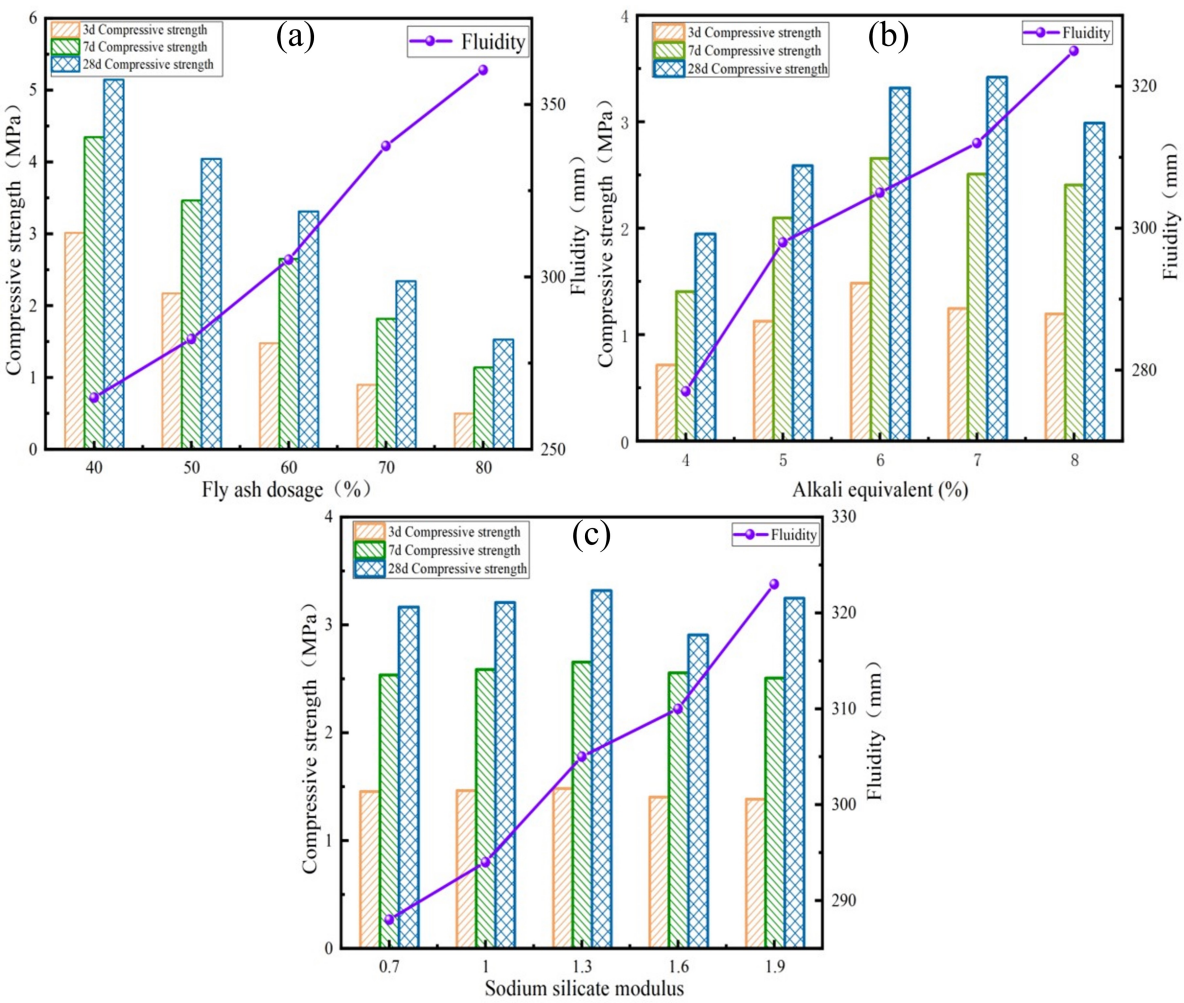
Results of single-factor experiments: (**a**) Fly ash dosage; (**b**) Alkali equivalent; (**c**) Sodium silicate modulus.

The test maintained an alkali equivalent of 6% and a sodium silicate modulus of 1.3, examining the influence of varying fly ash contents (40–80%) on the fluidity and compressive strength of cemented tailings backfill. Figure 5a illustrates that increasing the fly ash dosage from 40% to 80% results in a fluidity rise from 265 mm to 360 mm, reflecting a 35.8% increase. The large specific surface area and high reactivity of slag cause it to absorb significant water in the slurry, reducing free water content and lowering fluidity. Fly ash can replicate the morphological effects of glass beads, and its incorporation enhances fluidity. Figure 5a indicates that the 3-day, 7-day, and 28-day compressive strengths of the backfill decrease as the fly ash dosage increases. At a fly ash dosage of 40%, the 3-day compressive strength of the backfill was 3.02 MPa, while at 80% dosage, it dropped to 0.51 MPa, a reduction of 83.1%. This indicates that the fly ash dosage significantly affects the compressive strength of the backfill. At a fly ash dosage of 40%, the 7-day and 28-day compressive strengths were 4.35 MPa and 5.15 MPa, respectively. At an 80% dosage, these strengths were 1.15 MPa and 1.54 MPa, both higher than the 3-day strength. The increases in the 7-day and 28-day strengths were 44.0% and 70.5% at a 40% fly ash dosage, and 125.5% and 202.0% at 80%. Slag contributes to the early strength of the backfill, while the inclusion of fly ash reduces compressive strength but aids in the development of later strength. This is due to the higher calcium content and activity of slag, which promote the formation of early strength [32]; in the preliminary reaction, fly ash hydrolyzes less active material. As the hydration reaction progresses, fly ash is gradually hydrolyzed, generating more cementitious products and slowly increasing strength.

#### 3.1.2. Alkali Equivalent

The test established a fly ash dosage of 60% and a sodium silicate modulus of 1.3 to investigate the effects of alkali equivalent (ranging from 4% to 8%) on the fluidity and compressive strength of the cemented tailings backfill material. Figure 5b indicates that fluidity increases from 277 mm to 325 mm as the alkali equivalent rises from 4% to 8%. The increase in alkali equivalent promotes the breaking of chemical bonds, such as Ca-O, in the surface layer of particles and accelerates the dissolution process. Furthermore, an increase in alkali equivalent also implies a higher sodium silicate content, leading to a decrease in the dynamic yield stress of the slurry [33], which consequently enhances fluidity. As illustrated in Figure 5b, the compressive strength of the backfill initially increases and then decreases with rising alkali equivalent. The maximum compressive strength of the backfill occurred at an alkali equivalent of 6%, with compressive strengths of 1.49 MPa, 2.66 MPa, and 3.32 MPa at 3 days, 7 days, and 28 days, respectively. Compared to an alkali equivalent of 4%, the strengths increased by 106.9%, 88.65%, and 70.26%. This increase in alkali equivalent enhances the system’s alkalinity, promoting the breaking of Si-O and Al-O bonds in the surface layers of slag and fly ash particles. This generates significant amounts of [SiO_4_]^4−^ and [AlO_4_]^5−^, which participate in the hydration reaction to form C-S-H gels [34,35]. However, excessively high alkali equivalents cause the backfill compressive strength to decrease. This occurs because highly polymerized C-S-H gels occupy numerous nucleation sites, limiting the polymerization reaction [7,36]. Additionally, excess [SiO_4_]^4−^ and [AlO_4_]^5−^ inhibit the further development of the gel structure [37].

#### 3.1.3. Sodium Silicate Modulus

The test established a fly ash dosage of 60% and an alkali equivalent of 6% to investigate the effect of the sodium silicate modulus (ranging from 0.7 to 1.9) on the fluidity and compressive strength of the cemented tailings backfill. As illustrated in Figure 5c, the fluidity increased with the sodium silicate modulus, showing a rise of 12.2% when the modulus changed from 0.7 to 1.9. This increase in the sodium silicate modulus results in a higher concentration of [SiO_4_]^4−^ in the system, which reduces inter-particle stress and decreases collisions between particles, thereby enhancing fluidity [38]. Figure 5c indicates that the compressive strength initially increases and then decreases with the increasing sodium silicate modulus, reaching a maximum at a modulus of 1.3. When the sodium silicate modulus was reduced from 1.9 to 1.3, the compressive strengths at 3 days, 7 days, and 28 days improved by 7.2%, 5.98%, and 2.15%, respectively, indicating that decreasing the sodium silicate modulus enhances the early strength of the backfill. A lower sodium silicate modulus leads to a higher concentration of OH^−^ in the system. A higher concentration of OH^−^ promotes the activation of active ingredients in slag and fly ash, accelerating the formation of hydration products and resulting in improved early mechanical properties. A high-modulus sodium silicate solution slowly dissociates the powder in the early stages, but supplies a greater concentration of [SiO_4_]^4−^ for the subsequent hydration reaction. [SiO_4_]^4−^ acts as a reactant that combines with Ca^2+^ to form additional C-S-H gels, thereby enabling high-modulus sodium silicate to enhance the later strength of the backfill [39].

### 3.2. Results of Response Surface Methodology

#### 3.2.1. Experimental Results and Model Analysis

Seventeen groups of fit ratio tests were designed using BBD, with five groups serving as the centroids of the design area to estimate the test error. The design and experimental results of the response surface are presented in Table 4.

The quadratic polynomial regression equations for this model were established through the nonlinear fitting of the response surface test results, following the principle of least squares, as shown in Equations (1) and (2) as follows:

(1) $$Y_{1}=-21.98-0.26A+7.07B+19.41C+0.02\mathrm{AB}-0.10\mathrm{AC}-1.08\mathrm{BC}+0.002A^{2}-0.53B^{2}-2.84C^{2}$$

(2) $$Y_{2}=-497.35+2.5A+96.04B+475.28C-0.55\mathrm{AB}-2.08\mathrm{AC}-19.17\mathrm{BC}+0.055A^{2}-2.5B^{2}-80.6C^{2}$$

where $Y_{1}$ represents the 28-day compressive strength, $Y_{2}$ denotes fluidity, A indicates the fly ash dosage (%), B signifies the alkali equivalent (%), and C represents the sodium silicate modulus.

To further evaluate the accuracy and validity of the model, the regression model was analyzed using analysis of variance (ANOVA), and the results are presented in Table 5. Table 5 indicates that the *p*-values for both models are less than 0.0001, demonstrating that the regression models are both highly significant and statistically relevant. The *p*-values for the out-of-fit terms are all greater than 0.05, indicating that these terms are not significant and that the models are well fitted. In the 28-day compressive strength model, the order of significance for the factors is A > B > C, while the order for the interactions is BC > AC > AB. In the fluidity model, the order of significance for the factors is A > C > B, and the order for the interactions is AC > BC > AB. The $R^{2}$ values for the two models are 0.9878 and 0.9970, indicating a strong correlation; the adjusted $R^{2}$ values are 0.9721 and 0.9932, signifying that the models account for 97.21% and 99.32% of the variations in the response values, respectively. Furthermore, the Cv for both models is less than 10%, and the signal-to-noise ratio exceeds four, further indicating a good fit to the actual data [40]. These models are reliable and suitable for optimizing each parameter of the backfill material.

#### 3.2.2. Analysis of Fluidity

**Figure 6:**
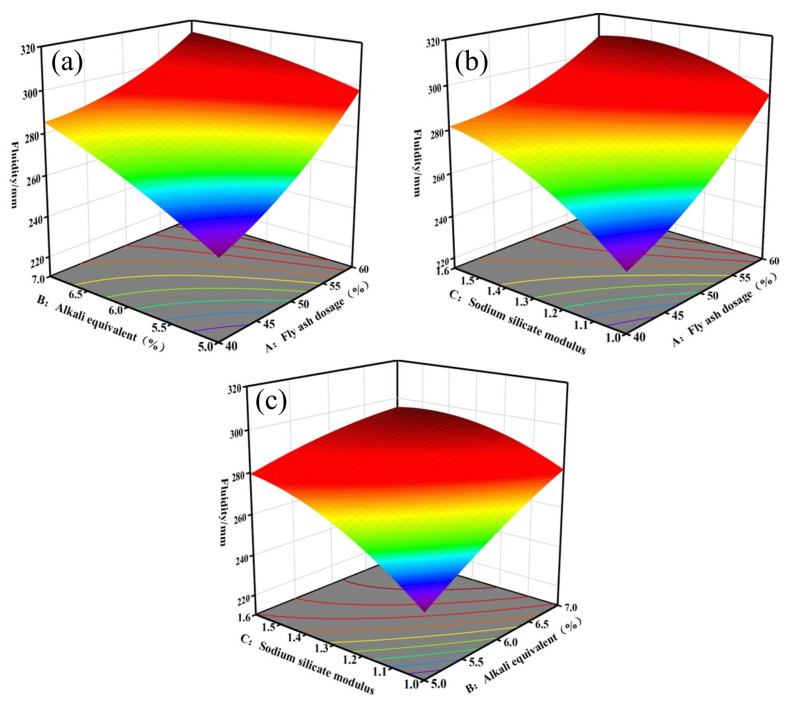
Response surface of fluidity models: (**a**) AB, (**b**) AC, (**c**) BC.

**Figure 7:**
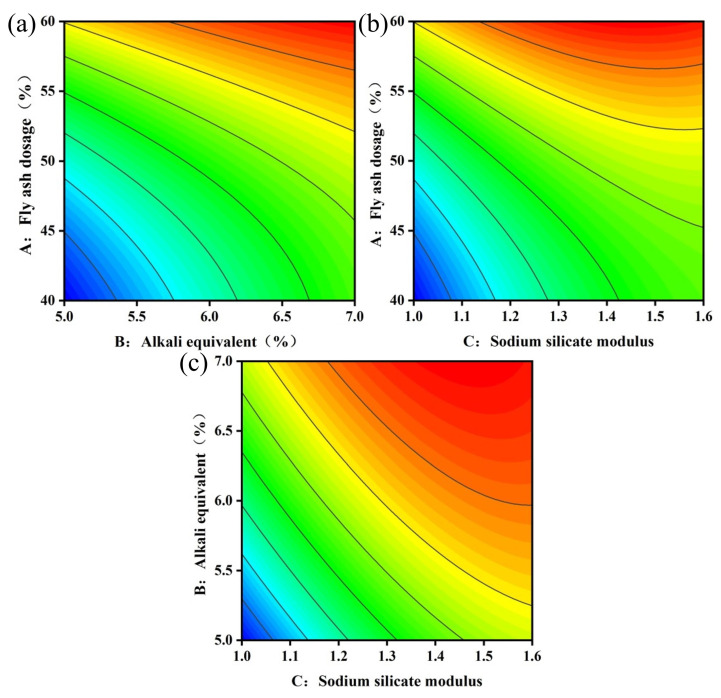
Contour maps of fluidity model: (**a**) AB, (**b**) AC, (**c**) BC.

Figure 6 and Figure 7 illustrate the response surface and contour maps depicting the two-by-two interactions of the fly ash dosage (A), alkali equivalent (B), and sodium silicate modulus (C) on fluidity. Figure 6 indicates that fluidity significantly improves with an increasing fly ash dosage and alkali equivalent, with the influence of the fly ash dosage being more pronounced. This occurs because the particle morphology of fly ash enhances slurry fluidity [41]. Reference [42] indicates that fluidity decreases by 17% when the slag content increases from 85% to 100%. Consequently, replacing slag with fly ash benefits slurry pumping. In conjunction with Figure 7, a more pronounced interaction between the fly ash dosage and sodium silicate modulus occurs when the fixed alkali equivalent is 6%. This interaction is illustrated in Figure 6b. At a fly ash dosage of 40%, fluidity increases as the sodium silicate modulus increases. This occurs because increasing the sodium silicate modulus raises the silicate ion concentration in the system and reduces inter-particle stress. The results are also supported by the findings of many researchers [33,43].

#### 3.2.3. Analysis of 28-Day Compressive Strength

**Figure 8:**
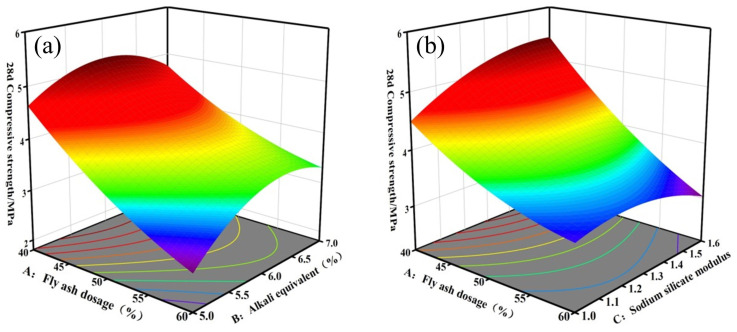

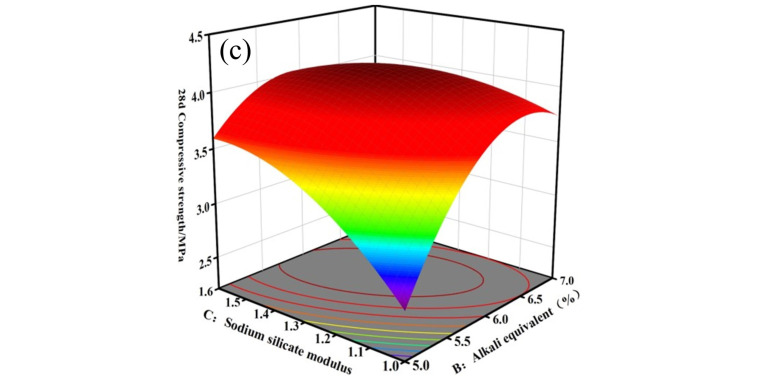
Response surface of 28 *d* compressive strength model: (**a**) AB, (**b**) AC, (**c**) BC.

**Figure 9:**
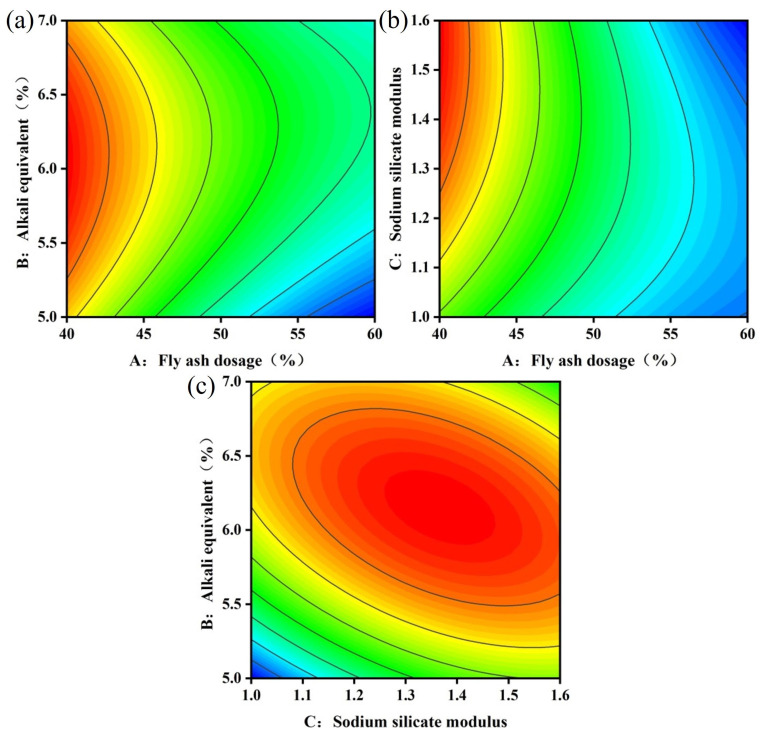
Contour maps of 28d compressive strength model: (**a**) AB, (**b**) AC, (**c**) BC.

Figure 8 and Figure 9 illustrate the response surface and contour maps depicting the two-by-two interactions of fly ash dosage (A), alkali equivalent (B), and sodium silicate modulus (C) on the 28-day compressive strength. Figure 8a illustrates the impact of the AB interaction on 28-day compressive strength at a sodium silicate modulus of 1.3. At an alkali equivalent of 5%, increasing the fly ash dosage from 40% to 60% resulted in a 42.6% reduction in compressive strength. High dosages of fly ash reduce cementitious products and subsequently decrease compressive strength. The compressive strength of the backfill reached higher values when the alkali equivalent ranged from 6% to 6.5%. The high alkaline environment accelerated the hydrolysis polymerization reaction and enhanced the polymerization of [SiO_4_]^4−^ and [AlO_4_]^5−^ with Ca^2+^, positively contributing to compressive strength development. Figure 8b illustrates the influence of the fly ash dosage and sodium silicate modulus on the 28-day compressive strength of the backfill at an alkali equivalent of 6%. At a fly ash dosage of 40%, the compressive strength initially increased before gradually decreasing with a higher sodium silicate modulus. This occurs because sodium silicate not only creates an alkaline environment for the polymerization reaction but also supplies reactive silica fractions as reactants, resulting in the formation of more hydration products [44]. The 28-day compressive strength of alkali-activated backfill material is significantly higher than that of conventional cement-based materials [45,46], thereby ensuring mining safety. Furthermore, the elliptical contours in Figure 9 demonstrate a significant interaction between the alkali equivalent and sodium silicate modulus, supporting the findings presented in Table 5.

#### 3.2.4. Validation of Optimal Ratio

The performance of the cemented tailings backfill material must meet the following specific project requirements: 28-day compressive strength ≥ 3 MPa and fluidity ≥ 220 mm. To reduce project costs while meeting these requirements, increasing the fly ash dosage is advantageous. The backfill material proportions were optimized using the numerical function in BBD to maximize strength, fluidity, and fly ash dosage. Five parallel tests were conducted to verify the accuracy of the recommended optimal ratios. The test results are presented in Table 6. Table 6 shows that the relative errors between the measured and model-predicted values for 28-day compressive strength and fluidity do not exceed 5%, indicating reliability. The optimal ratio was 47.8% fly ash, 6.10% alkali equivalent, and a sodium silicate modulus of 1.44.

### 3.3. Results of Dry–Wet and Freeze–Thaw Cycle Experiments

#### 3.3.1. Appearance Changes

**Figure 10:**
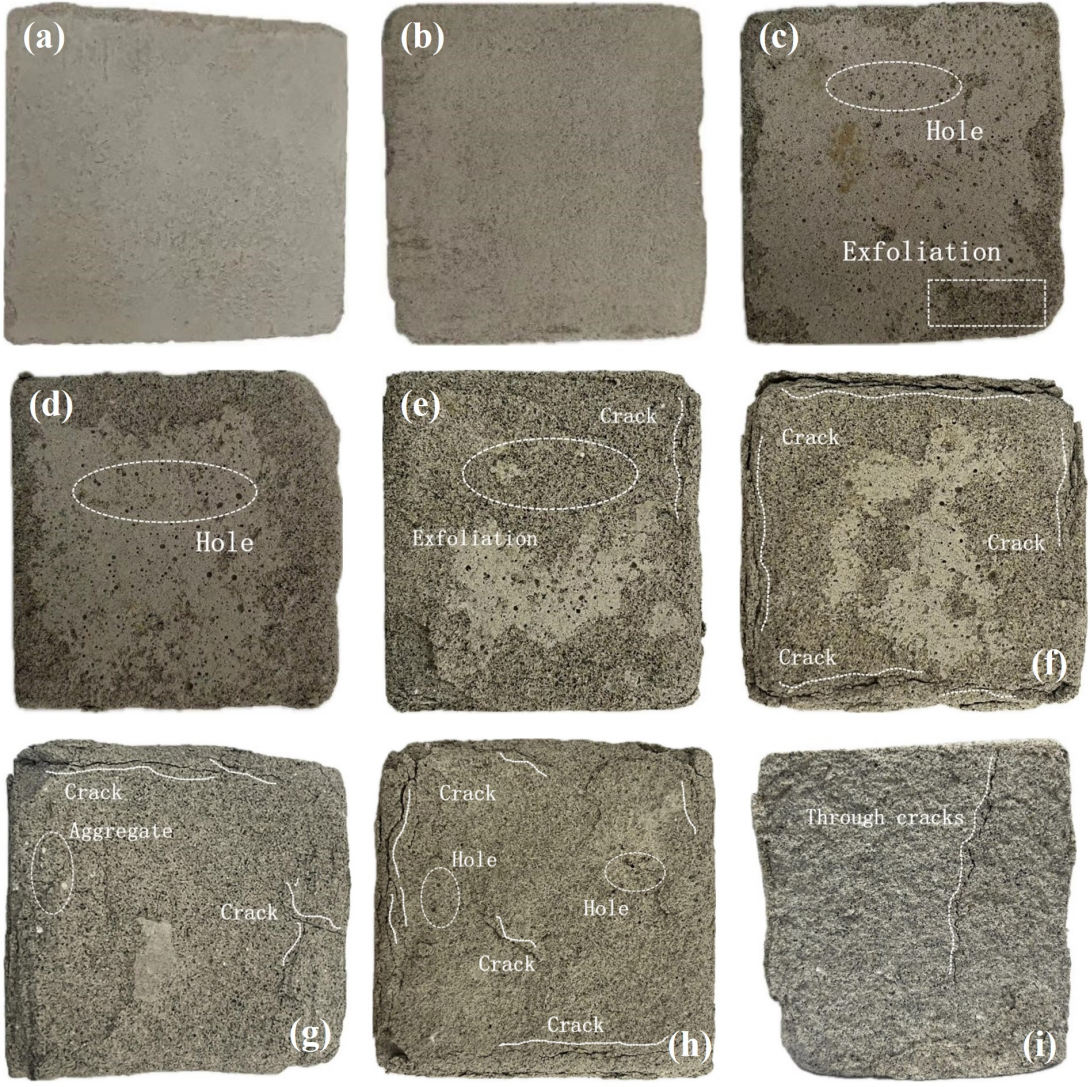
Appearance changes of backfill: (**a**) 0 cycles; (**b**) 1 cycle; (**c**) 3 cycles; (**d**) 5 cycles; (**e**) 7 cycles; (**f**) 9 cycles; (**g**) 11 cycles; (**h**) 13 cycles; (**i**) 15 cycles.

Figure 10 illustrates the appearance of the specimens subjected to various DW-FT cycles. Following the DW-FT cycles, the specimens exhibited the following changes in appearance:

- At the beginning of the cycle (0–3 cycles), the specimen exhibited the following changes: After one cycle, the skin remained intact, and the overall appearance was largely unchanged, showing no visible damage. After three cycles, however, the skin at the edges and corners began to deteriorate, with a few tiny holes appearing, although the specimen remained generally intact;
- In the middle of the cycle (5–9 cycles), the specimens exhibited notable changes: After five cycles, the surface holes enlarged, indicating slight damage. After seven cycles, the skin around the specimen completely detached, exposing the internal aggregate and resulting in small cracks at the edges. By nine cycles, cracks at the peripheries progressed steadily, eventually coalescing into through cracks. The outermost layer of the specimen’s skin appeared loose and began to fall off, rendering the specimen no longer intact;
- In the later stages of the cycle (11–15 cycles), cracks began to develop within the specimen: As the cycles increased, these cracks gradually progressed, ultimately forming through cracks. Concurrently, the internal aggregate became exposed. The specimen’s surface became uneven, and the exterior appeared loose and detached due to significant erosion from the DW-FT cycles.

#### 3.3.2. Quality and Strength Changes

**Figure 11:**
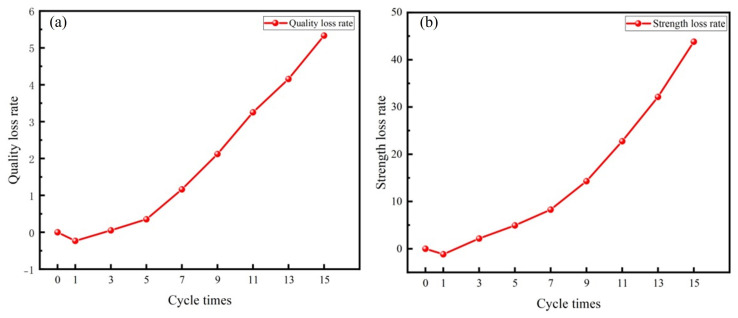
Changes of backfill: (**a**) quality and (**b**) strength.

Figure 11 illustrates the rates of quality and strength loss in the specimens subjected to varying numbers of DW-FT cycles.

As shown in Figure 11, after one cycle, the rates of quality and strength loss are negative, indicating a slight increase in the specimen’s quality and strength. However, with the increasing number of cycles, both quality and strength exhibited a decreasing trend. After 15 cycles, the specimens lost 5.3% of their quality and 43.8% of their strength. This may result from the specimen being in an unsaturated state during the pre−cycling period, allowing it to absorb significant amounts of water during immersion. Water molecules penetrate the specimen through pore channels, contact incompletely reacted active substances, and undergo secondary hydration reactions, producing hydration products such as AFt and C−S−H gels. The hydration products bond together within the specimen’s pores, resulting in a denser interior and increased quality and strength.

With the increasing number of cycles, the hydration reaction within the specimen gradually weakened, while the coupled erosion effects of DW−FT cycles intensified. Firstly, during the dry−wet cycle, changes in humidity primarily drive water migration within the specimen. During immersion, water enters the specimen through the pore channels. Water softens and dissolves the specimen, reducing the bonding effectiveness of the cementitious products. During drying, the specimen loses water and shrinks, leading to the formation of cracks. Subsequently, during the freeze−thaw cycle, temperature changes primarily affect the morphology of water molecules. The pore water within the specimen repeatedly freezes, causing the pores to expand in volume. Subsequently, cracks form within the specimen, severely damaging the backfill structure [47]. Additionally, the specimen’s external surface gradually peels away due to the erosive effects of DW−FT cycles. The specimen’s interior comes into contact with air, undergoing a carbonation reaction that reduces the strength of the backfill [48].

#### 3.3.3. X-ray Diffraction Analysis

**Figure 12:**
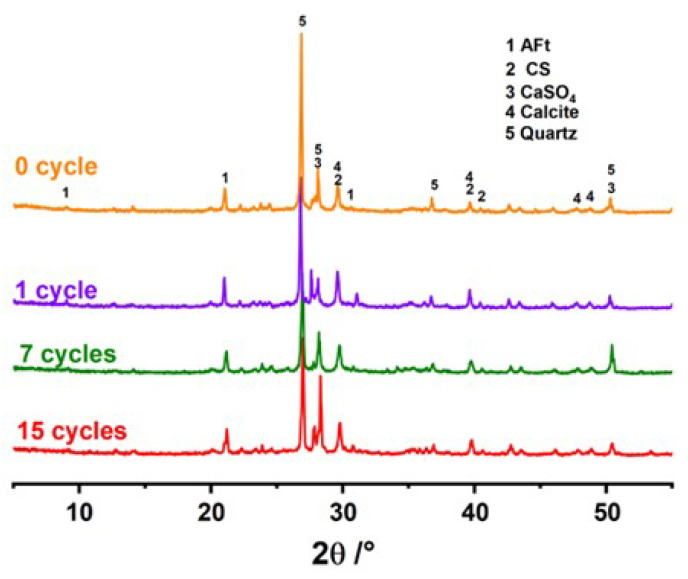
XRD of backfill.

XRD was employed to analyze the hydration products of samples subjected to zero, one, seven, and fifteen cycles, as illustrated in Figure 12. The main phases of the cemented tailings backfill material, as illustrated in Figure 12, are quartz, AFt, C-S-H gels (CS), gypsum (CaSO_4_), and calcite. Among these, quartz is inherent to the raw material, AFt and other phases are products of the hydration reaction, while calcite results from carbonation. After one cycle, the diffraction peaks of quartz weakened, while those of AFt and C-S-H gels were enhanced. This indicates that the presence of incompletely reacted slag and fly ash at the beginning of the cycle, along with contact with water molecules, promoted secondary hydration reactions within the specimen. The increase in AFt is the main contributor to the enhanced strength of the backfill. This conclusion is also corroborated by the findings of Mohammed et al. [49]. As the number of cycles increased, the diffraction peak of AFt continued to weaken. This indicates that the gelling products within the specimen gradually diminished due to repeated dissolution by water. Notably, a specific variation in the XRD is the increasing diffraction peaks of calcite. Damage to the pore structure accelerated the carbonation process, leading to a gradual increase in calcite content, and a similar phenomenon was observed in dry and wet cycling tests [48].

#### 3.3.4. Scanning Electron Microscopy–Energy Dispersive Spectroscopy Analysis

**Figure 13:**
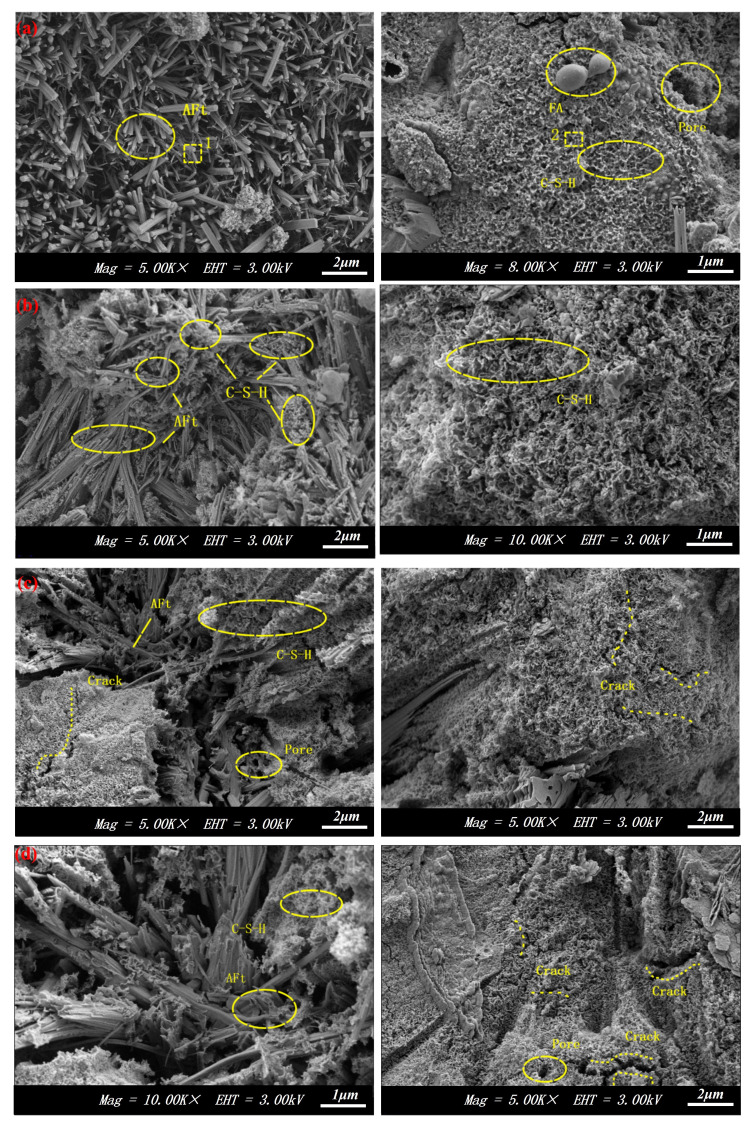
SEM of backfill: (**a**) 0 cycles; (**b**) 1 cycle; (**c**) 7 cycles; (**d**) 15 cycles.

**Figure 14:**
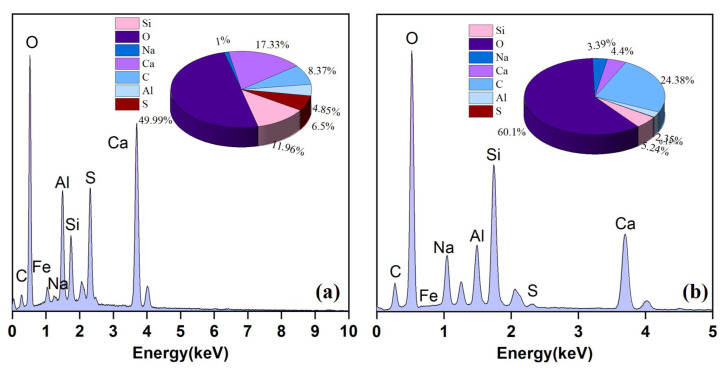
EDS spectrum analysis: (**a**) Location 1 in Figure 13a; (**b**) Location 2 in Figure 13a.

SEM-EDS was employed to further analyze the micro-morphology and hydration products of the samples subjected to zero, one, seven, and fifteen cycles, as shown in Figure 13 and Figure 14. Figure 13a shows that the hydration products in the backfill mainly consist of numerous needle-like and flocculent gels. Combined with the energy spectrum analysis in Figure 14, these hydration products are dominated by AFt and C-S-H gels. Additionally, a small number of spherical fly ash particles and pores were observed in the specimens, indicating insufficient hydration during the pre-cycle period. After one cycle, a notable increase in the contents of AFt and C-S-H gels was observed within the specimens, along with a reduction in pore structure. The interweaving and overlapping of the hydration products resulted in a denser internal structure, confirming the enhancement of the compressive strength and quality of the backfill. However, after seven cycles, a decrease in the contents of AFt and C-S-H gels was observed in the specimens, along with a transition of the internal structure from dense to loose and the emergence of tiny pores and cracks. After 15 cycles, the structure of the specimens exhibited significant dispersion, forming plate-like structures of varying sizes. The internal pores were enlarged, and the cracks increased significantly.

A comparison of the microscopic morphology of the backfill at different cycle times revealed a gradual reduction in hydration products and a weakening of adhesion between particles due to the repeated erosion and freezing of pore water. Concurrently, the number of pores increased and the pore volume gradually expanded. The formation of continuous cracks caused the backfill to split into plate-like bodies, leading to significant reductions in its macroscopic strength. Numerous researchers have reached similar conclusions in their studies on backfill durability [20,50].

#### 3.3.5. Nitrogen Adsorption Test Analysis

**Figure 15:**
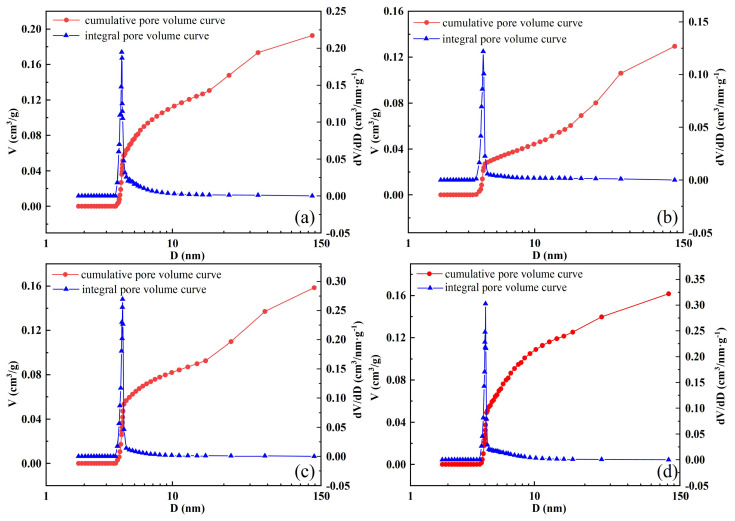
Pore distribution curve of backfill: (**a**) 0 cycle; (**b**) 1 cycle; (**c**) 7 cycles; (**d**) 15 cycles.

The NAT was employed to analyze the pore characteristics of samples subjected to zero, one, seven, and fifteen cycles. The pore distribution curve is presented in Figure 15. Figure 15 shows that the integral pore volume curve of the specimen resembles a single− peaked curve. A distinct peak is observed between pore sizes of 3.5 nm and 4 nm. The peak values of the integral pore volume for each specimen are 0.1947, 0.1219, 0.2696, and 0.3028, respectively. As the number of cycles increases, the peak value of the integral pore volume initially decreases and then increases. This indicates that the hydration products formed at the beginning of the cycle filled the internal pores of the specimen, leading to a reduction in the pore volume. Subsequently, the internal pores of the specimen increased due to the combined effects of dry−wet and freeze−thaw cycling, which diminished the macroscopic compressive strength of the backfill.

The cumulative pore volume curve of the backfill is divided into three stages. The first stage (0 < d < 2 nm) corresponds to microporous pores. The second stage (2 nm < d < 50 nm) corresponds to mesopores. The final stage (d > 50 nm) corresponds to macropores. Figure 15 shows that the pore composition in the backfill is predominantly composed of mesopores and macropores, with a minor presence of micropores.

**Figure 16:**
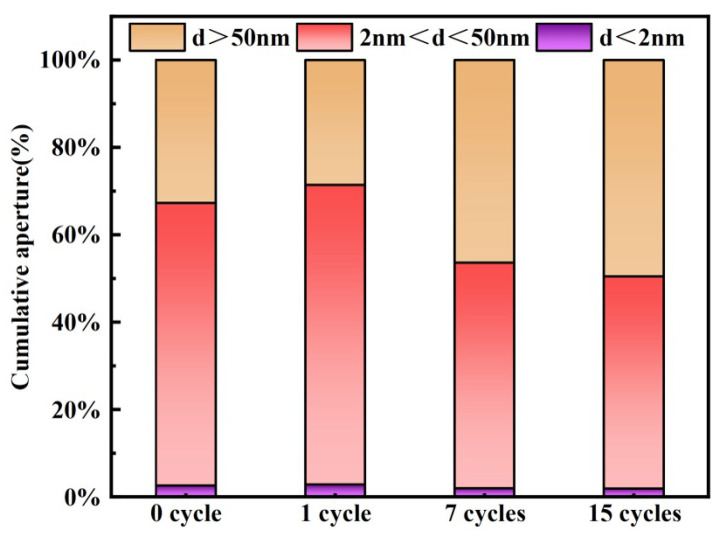
The cumulative pore size histogram of the backfill.

Large pores (d > 50 nm) are classified as harmful pores, medium pores (2 nm < d < 50 nm) as less harmful pores, and micropores (0 nm < d < 2 nm) as harmless pores [51]. Figure 16 presents the cumulative pore size histogram of the backfill for varying numbers of cycles. Harmless pores are less affected by the number of cycles. However, as the number of cycles increases, the harmful pores in the backfill initially decrease and then increase. This indicates that during the later stages of cycling, the combined effects of dry−wet and freeze−thaw cycling can significantly increase harmful pores in the backfill, thereby reducing its compressive strength.

## 4. Conclusions

In this study, cemented tailings backfill was prepared using alkali-activated slag and fly ash as primary raw materials. RSM was developed for the fluidity and 28-day compressive strength of the backfill, and the optimal ratio was determined. Additionally, XRD, SEM-EDS, and NATs were employed to investigate the micro-mechanisms of the backfill under the combined effects of dry–wet and freeze–thaw cycling. The main conclusions of the study are as follows:

1. The R^2^ values for the fluidity and 28-day compressive strength models obtained via RSM were 0.9970 and 0.9878, respectively, with P-values both below 0.0001. Both models demonstrate high reliability and are suitable for predicting backfill performance. Response surface analysis revealed that the factors influencing fluidity were ranked as fly ash dosage > sodium silicate modulus > alkali equivalent, with the interaction between the fly ash dosage and sodium silicate modulus being the most significant. The factors influencing 28-day compressive strength were ranked as fly ash dosage > alkali equivalent > sodium silicate modulus, with the interaction between the alkali equivalent and sodium silicate modulus being the most significant;
2. The optimal ratio derived from the RSM is 47.8% fly ash dosage, 6.10% alkali equivalent, and a sodium silicate modulus of 1.44. At this ratio, the backfill exhibited a fluidity of 280 mm and a 28-day compressive strength of 4.4 MPa, achieving optimal overall performance;
3. The macroscopic behavior of the backfill under the combined effects of wet–dry and freeze–thaw cycling is as follows: pore initiation → pore expansion → crack formation → crack propagation → structural damage. The strength of the backfill exhibited a continuous decrease following a minor increase. XRD and SEM-EDS analyses indicated that the primary hydration products of the specimens were flocculent C-S-H gels and needle-like AFt. As the number of cycles increases, the gradual reduction of gelling products within the specimens and the ongoing deterioration of the pore structure are the primary reasons for the decrease in strength;

This study focuses on the management of goafs by preparing a low-carbon, low-cost cemented tailings backfill from solid waste materials and investigating its durability under DW-FT cycles. However, the diverse sources and varying compositions of solid waste materials may result in different properties for materials prepared using identical formulations. This paper does not adequately address material design. Furthermore, future research should investigate the sulfate and chloride resistance, as well as the environmental compatibility, of alkali-activated backfill material. Additionally, AI-based detection and design methods offer numerous concepts for our future research, including physical information networks [52], deep learning techniques [53], and generative adversarial networks [54,55].

## Figures and Tables

**Table 1:** Main chemical composition of raw materials (wt%).

Composition	SiO_2_	Al_2_O_3_	CaO	MgO	Fe_2_O_3_	Na_2_O	SO_3_
Slag	28.50	15.12	42.20	6.73	0.62	0.33	2.41
Fly ash	56.21	24.75	1.63	0.92	4.70	0.51	0.36
Iron tailings	51.21	14.02	7.40	4.25	11.91	1.50	0.20

**Table 2:** Design of single-factor experiments.

Constant Factors		Control Factor
Alkali equivalent	Sodium silicate modulus	Fly ash dosage
6%	1.3	40%, 50%, 60%, 70%, 80%
Fly ash dosage	Sodium silicate modulus	Alkali equivalent
60%	1.3	4%, 5%, 6%, 7%, 8%
Fly ash dosage	Alkali equivalent	Sodium silicate modulus
60%	6%	0.7, 1.0, 1.3, 1.6, 1.9

**Table 3:** Response surface factor and level design.

Level	A/wt%	B/wt%	C
−1	40	5	1.0
0	50	6	1.3
1	60	7	1.6

**Table 4:** Design and experimental results of response surface.

Sample	Factor A/%	Factor B/%	Factor C	Fluidity/mm	28 d Compressive Strength/MPa
1	40	5	1.3	249	4.51
2	60	5	1.3	298	2.59
3	40	7	1.3	285	4.65
4	60	7	1.3	312	3.42
5	40	6	1.0	240	4.61
6	60	6	1.0	294	3.21
7	40	6	1.6	281	5.52
8	60	6	1.6	310	2.91
9	50	5	1.0	239	2.62
10	50	7	1.0	280	3.71
11	50	5	1.6	278	3.61
12	50	7	1.6	296	3.41
13	50	6	1.3	282	4.05
14	50	6	1.3	284	4.04
15	50	6	1.3	282	4.17
16	50	6	1.3	283	4.22
17	50	6	1.3	284	4.11

**Table 5:** Analysis of variance in the response surface regression model.

Source of Variance	DF	Fluidity			28 d Compressive Strength		
		Mean Square	F Value	*p* Value	Mean Square	F Value	*p* Value
Model	9	775.92	261.76	<0.0001	1.06	63.02	<0.0001
A	1	3160.13	1066.07	<0.0001	6.41	380.37	<0.0001
B	1	1485.13	501.01	<0.0001	0.4325	25.67	0.0015
C	1	1568.00	528.96	<0.0001	0.2112	12.54	0.0095
AB	1	121.00	40.82	0.0004	0.1190	7.06	0.0326
AC	1	156.25	52.71	0.0002	0.3660	21.73	0.0023
BC	1	132.25	44.61	0.0003	0.4160	24.69	0.0016
A^2^	1	127.37	42.97	0.0003	0.1680	9.97	0.0160
B^2^	1	26.32	8.88	0.0205	1.16	68.95	<0.0001
C^2^	1	221.32	74.66	<0.0001	0.2743	16.28	0.0050
Residual	7	2.96	-	-	0.0168	-	-
Lack of fit	3	5.58	5.58	0.0650	0.0314	5.25	0.0715
Pure error	4	1.0000	-	-	0.0060	-	-
R^2^		0.9970			0.9878		
Adj R^2^		0.9932			0.9721		
Perd R^2^		0.9608			0.8406		
C_*v*_/%		0.613			3.38		
Adeq Precision		56.1344			29.4326		

**Table 6:** Prediction model experimental validation results.

Sample	A/%	B/%	C	28 d Compressive Strength			Fluidity		
				Predicted Value/MPa	Actual Value/MPa	Error/%	Predicted Value/MPa	Actual Value/MPa	Error/%
1	47.9	6.10	1.44	4.37	4.40	0.68	285	280	1.79
2				4.37	4.43	1.35	285	288	1.04
3				4.37	4.32	1.16	285	292	2.40
4				4.37	4.41	0.91	285	280	1.79
5				4.37	4.33	0.92	285	277	2.89

## Data Availability

The original contributions presented in the study are included in the article, further inquiries can be directed to the corresponding author.

## Abbreviations

The following abbreviations are used in this manuscript:

AAM	alkali-activated materials
ANOVA	analysis of variance
BBD	Box–Behnken design
BJH	Barret–Joyner–Halenda
DW-FT	dry–wet and freeze–thaw
EDS	energy dispersive spectroscopy
NAT	nitrogen adsorption test
NMR	nuclear magnetic resonance
RSM	response surface methodology
SEM	scanning electron microscopy
XRD	X-ray diffraction
XRF	X-ray fluorescence
